# The Use of a Martius Flap in a Complex Pelvic Trauma Following Bone Fragment Removal from Deep within the Pelvis

**DOI:** 10.51894/001c.144423

**Published:** 2025-09-30

**Authors:** Gabriella Audia

**Affiliations:** 1 College of Osteopathic Medicine Michigan State University

## 65

### INTRODUCTION

The Martius flap was first described by Dr. Martius in 1928 when he used a labial flap of bulbocavernosus muscle for a urethrovaginal fistula repair. In urologic vaginal procedures, the Martius labial fat pad flap is routinely used in the periurethral or perivesical fascia when tissue integrity is a concern. Unstable pelvic fractures, which are defined as a break in the pelvis at two or more points, are most commonly caused by high energy blunt trauma. The objective of our study is to describe a unique case where a Martius flap was utilized for tissue coverage following a large polytrauma requiring bone fragment removal from deep within the pelvis.

### CASE DESCRIPTION

A 50-year-old female presented to the emergency department after a collision with a pickup truck while on a motorcycle. Initial imaging included a pelvic x-ray and an abdominal/pelvic computed tomography found a vertical comminuted and displaced fracture of the left superior and inferior pubic rami. Physical examination revealed intact vaginal mucosa. However, the left inferior pubic rami was palpable through the vaginal wall.

She was taken to the operating room. A 4 cm midline labia majora incision was made and cautery was then used to take down the subcutaneous tissues until the labial fat pad was observed. The labial fat pad was then mobilized and retracted medially. Dissection was carried down through an avascular plane towards the inguinal crease. A 3.5cm bone fragment was isolated and removed. The previously mobilized labial fat pad was then interposed over the area of bone fragment removal and secured in place with 2-0 PDS. The patient did well post-operatively and had no vaginal pain or urinary issues.

### DISCUSSION/CONCLUSIONS

This case represents the first usage of a Martius flap for tissue coverage after the removal of a pelvic bone fragment. Following dissection and extrapolation of the fragment through the left labia, a Martius flap was then utilized to allow for proper tissue coverage to both fill the dead space adjacent to the vaginal wall and bladder and with the objective to prevent any future fistula formation.

